# Demethylzeylasteral inhibits cell proliferation and induces apoptosis through suppressing MCL1 in melanoma cells

**DOI:** 10.1038/cddis.2017.529

**Published:** 2017-10-26

**Authors:** Yuzu Zhao, Jiang He, Jun Li, Xingzhi Peng, Xianxing Wang, Zhen Dong, Erhu Zhao, Yaling Liu, Zonghui Wu, Hongjuan Cui

**Affiliations:** 1 State Key Laboratory of Silkworm Genome Biology, Southwest University, Chongqing 400715, China; 2 Department of Dermatology, the Third Hospital of Hebei Medical University, Shijiazhuang 050051, China; 3 Hospital of Southwest University, Southwest University, Chongqing 400716, China

## Abstract

Demethylzeylasteral is one of the extracts of *Tripterygium wilfordii* Hook F, which plays important roles in multiple biological processes such as inflammation inhibition, as well as immunosuppression. However, anti-cancer function and the underlying mechanisms of demethylzeylasteral in melanoma cells remain unclear. In this study, we demonstrate that demethylzeylasteral has an anti-tumor property in melanoma cells. Demethylzeylasteral not only inhibits cell proliferation through cell cycle arrest at S phase, but also induces cell apoptosis in melanoma cells. MCL1 is an anti-apoptotic protein in BCL2 family, and amplifies frequently in multiple human cancers. MCL1 is also known as a potential contributor for the resistance of BCL2 inhibitors, as well as various chemotherapeutic drugs. MCL1 is, therefore, regarded as a potential target for cancer therapy. Here, for the first time, we unveil that demethylzeylasteral suppresses the expression of MCL1. Interestingly, MCL1 interacts with S phase-related protein CDK2, and thereby inhibits it’s ubiquitin-dependent degradation. Together, demethylzeylasteral is a promising anti-tumor compound in melanoma cells. Demethylzeylasteral is also a potential inhibitor of MCL1.

Melanoma is also called malignant melanoma originating from melanocytes.^1^ Surgical resection is the main method for patients suffering early-stage melanoma.^1, 2^ Unfortunately, melanoma lesions always remain undetectable,^3^ which results in the delay for melanoma therapy.^4, 5^ Moreover, melanoma can break out at later stages,^6^ when melanoma cells disseminate to numerous organs, such as brain, lung or liver.^2^ Consequently, surgical operation is less favorable for patients. Chemotherapeutic therapy plays an important role in this case. In theory, chemotherapeutic agents can be transported everywhere through circulation.^7^ Nevertheless, current chemotherapeutic drugs fail to make significant effects. Even worse, melanoma cells are resistant to various chemotherapeutic agents because of its intrinsic resistance to apoptosis.^8, 9, 10, 11, 12^ Therefore, it is urgent to exploit some efficient chemotherapeutic drugs for melanoma treatment. Apoptosis activation can be regarded as a project to kill melanoma cells; therefore, anti-apoptotic and pro-apoptotic factors from intrinsic apoptosis pathways become potential targets for chemotherapeutic drugs.^11, 13^

B-cell CLL/lymphoma 2 (BCL2) family plays important roles in apoptosis regulation and are essential for cell death and survival determination.^14^ BCL2 is the first apoptotic regulator identified as an oncogene.^15^ After identification of BCL2, other BCL2 family members such as BCL2-like 1 (BCL- X_L_), myeloid leukemia 1 (MCL1), BAX and BAK were subsequently identified.^16^ According to four conserved BCL2 homology (BH) domains,^17^ BCL2 family comprises three main groups. BCL2, BCL-X_L_ and MCL1 belong to pro-survival group.^18, 19, 20^ The multiregion pro-apoptotic group containing BH1-3 domains include BAX and BAK. BIM, NOXA and PUMA only contain a BH3 domain,^17, 21, 22, 23, 24, 25^ therefore term to pro-apoptotic group.

BCL2 family members act as therapeutic targets.^26^ Over the past decades, numerous inhibitors of these proteins have been generated. ABT-737 is the first BH3 mimetic^27^ discovered as an inhibitor for BCL2, BCL-X_L_ and BCL-W.^28^ Then, the analogue of ABT-737, ABT-263 (Navitoclax) has been created.^29^ Since ABT-737 and ABT-263 were disclosed, many other dual inhibitors of BCL2 and BCL-X_L_, such as BM-1197 and S44563 have been developed.^30, 31^ Subsequently, various inhibitors selectively towards mono-protein have been reported. BCL2-selective inhibitor ABT-199 (also known as Venetoclax) has been developed.^32^ S55746 (also called BCL201 or Servier-1) is the second selective BCL2 inhibitor.^26^ Selective inhibitors of BCL-X_L_ have subsequently been reported, including WEHI-593, A-1155463 and A-1331852.^33, 34, 35^ Despite the generation of a great number of specific inhibitors, actual therapy remains still ineffective in the majority of the cases. Indeed, tumors result to be resistant to these chemotherapeutic agents mainly because of the expression of MCL1.^36, 37^

MCL1, which is overexpressed in many cancers, is another important pro-survival protein in BCL2 family.^20^ There are some MCL1-dependent tumors, such as breast cancer, acute myelocytic leukemia (AML) and non-small cell lung cancer (NSCLC).^38, 39, 40, 41^ Upon these cancers, BCL2 or BCL-X_L_ inhibitors did not work well. Besides, more and more studies indicated that MCL1 is a main contributor for resistance of various chemotherapeutic drugs, such as Taxol (TAX), Gemcitabine and Vincristine.^42, 43, 44^ Therefore, the generation of some compounds for MCL1 inhibitionis urgent. It is not hard to find that inhibitors mentioned above did dually or individually inhibit BCL2 and BCL-X_L_, but not MCL1.^26^ These BCL2 or BCL-X_L_ inhibitors constantly display very low affinity to MCL1, and therefore have no effects on MCL1 inhibition. Certainly, there are some MCL1 inhibitors, including UMI-77, A-1210477 and S63845.^45, 46, 47^ Nevertheless, there are numerous difficulties for the clinical application of these inhibitors^48^ as well. For example, there is little single-agent activity of S63845 in solid tumors; S63845 binds human MCL1 with greater affinity than murine MCL1. Some new inhibitors of MCL1 are still necessary to be generated. In this study, demethylzeylasteral, an extract of *Tripterygium wilfordii* Hook F,^49^ is proved to inhibit cell proliferation as well as inhibit MCL1 expression in melanoma cells. Besides, MCL1 serves as a regulator of cell cycle arrest and apoptosis induced by demethylzeylasteral. These findings indicate that demethylzeylasteral possesses an anti-cancer property in melanoma cells. Moreover, this study will enrich information for further investigations of MCL1 inhibitors.

## Results

### Demethylzeylasteral inhibits cell proliferation in melanoma cells

**Figure 1:**
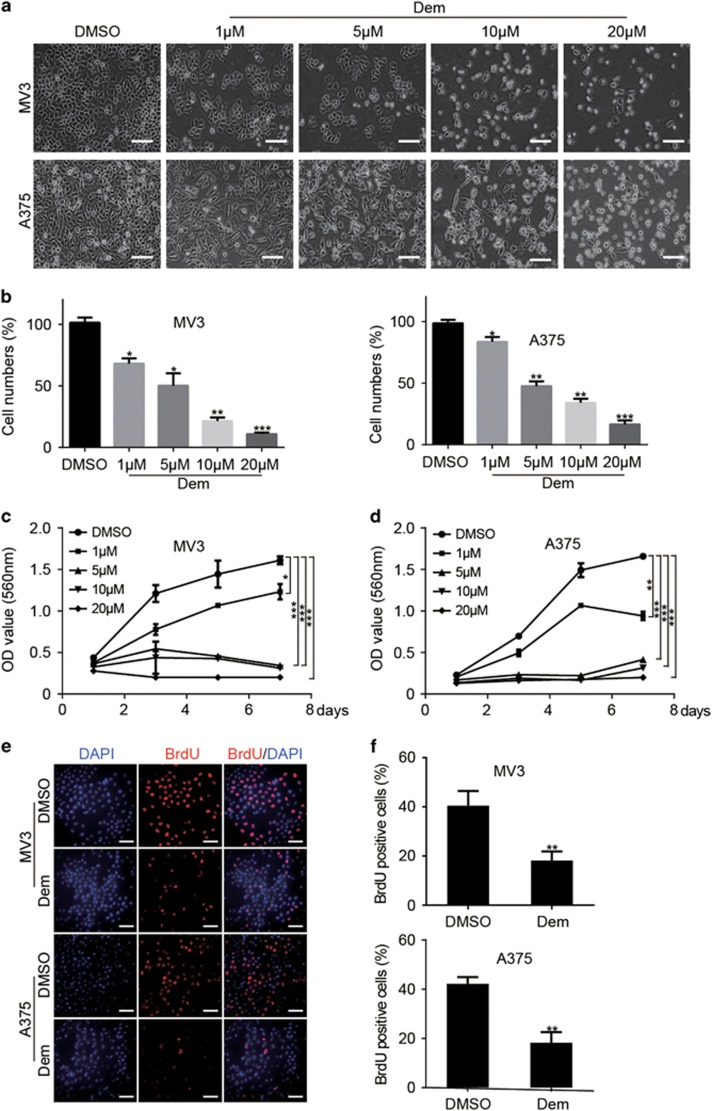
Demethylzeylasteral inhibits cell proliferation in melanoma cells. (**a**) The morphology of MV3 and A375 cells treating with demethylzeylasteral in different concentration of 1, 5, 10 and 20 *μ*M for 48 h. DMSO was used as control, scale bar=100 *μ*m. (**b**) Cell numbers of MV3 and A375 cells were counted and then displayed. Cell numbers of DMSO-treated group were regarded as 100%. (**c**,**d**) Viability of MV3 and A375 cells after treating with 1, 5,10 and 20 *μ*M demethylzeylasteral. DMSO was used as control. (**e**) BrdU-positive MV3 and A375 cells after treating with 5 *μ*M demethylzeylasteral for 48 h. DMSO was used as control, scale bar=100 *μ*m. (**f**) Quantification of BrdU-positive MV3 and A375 cells in (**e**). DMSO was used as control. All experiments were repeated at least three times. All data were used as mean±S.D., significant difference was tested by student’s *t*-test. *P<0.05, **P<0.01, ***P<0.001, *P*-value <0.05 were considered as statistically significant

In order to investigate the effects of demethylzeylasteral on melanoma cells, we treated melanoma cell lines, MV3 and A375, with different concentrations of demethylzeylasteral (1, 5, 10 and 20 *μ*M, dimethyl sulfoxide (DMSO) was used as control) for 48 h. Observing by microscopy, MV3 and A375 cells exposing to demethylzeylasteral showed significant morphological changes, cell numbers decreased in a dose-dependent manner as well (Figures 1a and b). 3-(4, 5-dimethylthiazol-2-yl)-2, 5-diphenyltetrazolium bromide (MTT) and bromodeoxyuridine (BrdU) assays were used to analyze cell growth and proliferation. MTT assay showed that comparing with DMSO group, melanoma cells treating with 5, 10 and 20 *μ*M demethylzeylasteral showed sharp declines in the growth curve. Actually, 5 *μ*M demethylzeylasteral showed similar effects as the concentration of 10 and 20 *μ*M. Cells treating with 1 *μ*M demethylzeylasteral showed decline in the growth curve as well; nevertheless, it is quite slight then declines induced by indicated concentrations mentioned above (Figures 1c and d). In consideration of the potential toxicities of demethylzeylasteral, we chose 5 *μ*M demethylzeylasteral as an indicated concentration for further investigations. BrdU assay showed that DNA synthesis reduced in the group treating with 5 *μ*M demethylzeylasteral for 48 h as well (Figures 1e and f).

### Demethylzeylasteral causes cell cycle arrest at S phase

**Figure 2:**
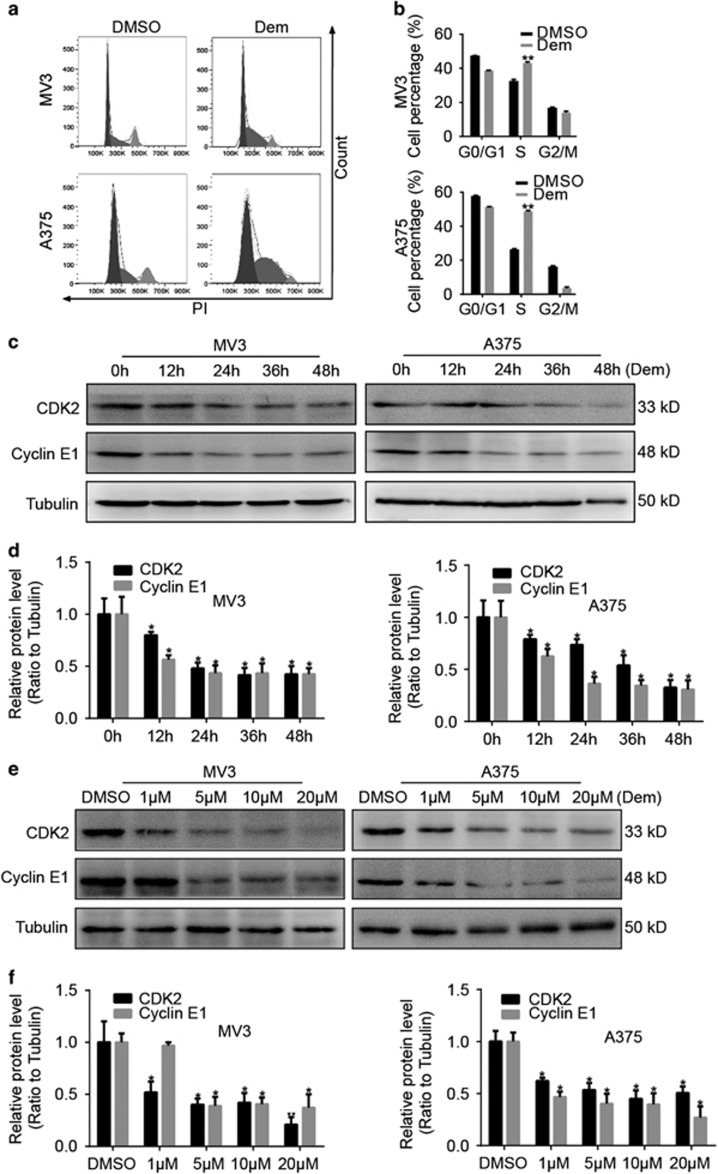
Demethylzeylasteral induces cell cycle arrest at S phase. (**a**) Cell cycle of MV3 and A375 cells treating with 5 *μ*M demethylzeylasteral for 24 h were analyzed by flow cytometry. DMSO was used as control. (**b**) Percentage of indicated MV3 and A375 cells in different phase. (**c**) The expression of CDK2 and Cyclin E1 in melanoma cells exposing to 5 *μ*M demethylzeylasteral in different time for 0, 12, 24 and 48 h. Tubulin was used as internal reference. (**d**) Densitometry of western blot in panel (**c**). (**e**) The expression of cell cycle-related proteins, CDK2 and Cyclin E1 in cells treated with demethylzeylasteral in different concentration of 1, 5, 10 and 20 *μ*M for 48 h. DMSO was used as control. Tubulin was used as internal reference. (**f**) Densitometry of western blot in panel (**e**). All experiments were repeated at least three times. All data were used as mean±S.D., significant difference was tested by student’s *t*-test. *P<0.05, **P<0.01, ***P<0.001, *P*-value <0.05 were considered as statistically significant

We further checked cell cycle to evaluate whether demethylzeylasteral inhibits cell proliferation by causing cell cycle arrest. The results showed that the percentages of S phase in both MV3 and A375 cells were dramatically increased following 5 *μ*M demethylzeylasteral treatment for 24 h (Figures 2a and b),which means demethylzeylasteral causes cell cycle arrest at S phase. To certify these results, we examined CDK2 and Cyclin E1 proteins which are essential for G1/S phase transition. After treating melanoma cells with demethylzeylasteral in concentration of 5 *μ*M for 0, 12, 24, 36 and 48 h. The results showed that CDK2 and CyclinE1 decreased in a time-dependent manner (Figures 2c and d). We then dosed melanoma cells with different concentrations of demethylzeylasteral (1, 5, 10 and 20 *μ*M, DMSO was used as control) for 48 h; we found that CDK2 and Cycin E1 decreased in a dose-dependent manner (Figures 2e and f).

### Demethylzeylasteral induces apoptosis in melanoma cells

**Figure 3:**
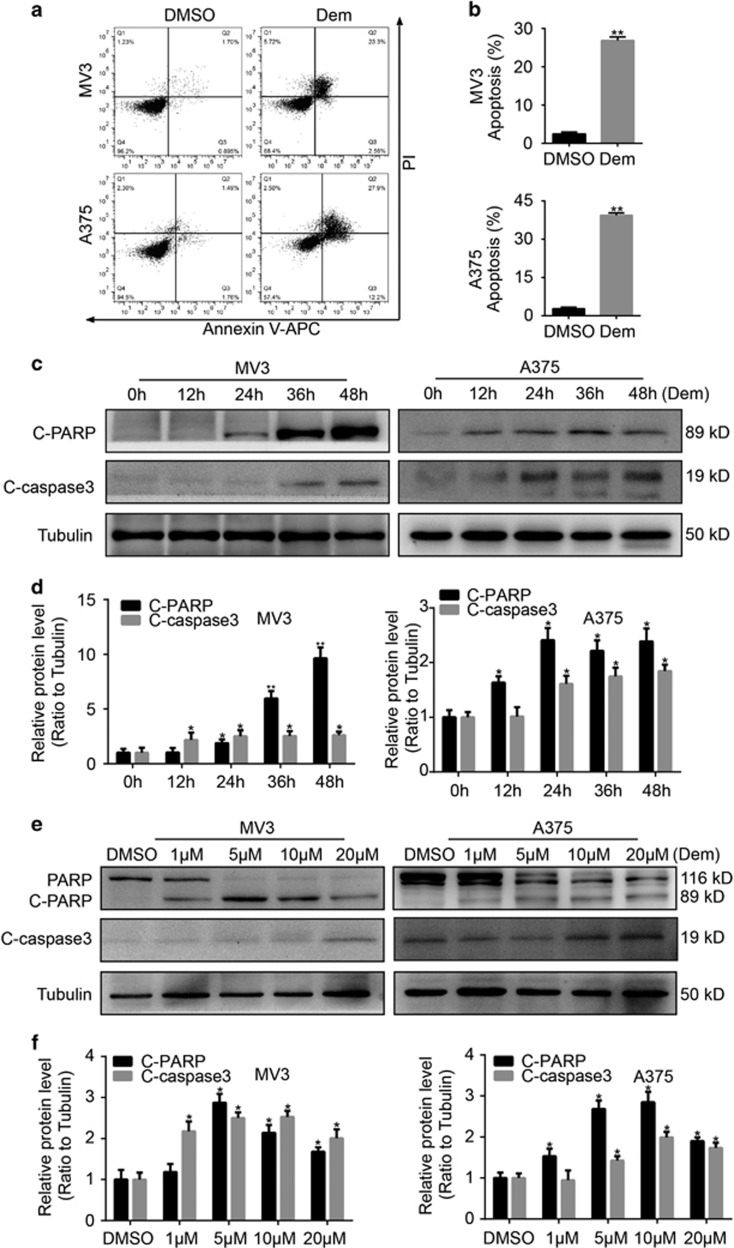
Demethylzeylasteral causes apoptosis in melanoma cells. (**a**) apoptosis of MV3 and A375 cells treating with 5 *μ*M demethylzeylasteral for 48 h were examined using flow cytometry. DMSO was used as control. (**b**) Apoptotic rate of MV3 and A375 cells in panel (**a**) was quantified. (**c**) The expression of apoptotic proteins, cleaved-PARP and cleaved-caspase3 in cells treated with 5 *μ*M demethylzeylasteral in different time for 0, 12, 24 and 48 h. Tubulin was used as internal reference. (**d**) Densitometry of western blot in panel (**c**). (**e**) The expression of apoptotic proteins, PARP and cleaved-caspase3 in cells treated with demethylzeylasteral in different concentration of 1, 5,10 and 20 *μ*M for 48 h. DMSO was used as control. Tubulin was used as internal reference. (**f**) Densitometry of western blot in panel (**e**). All experiments were repeated at least three times. All data were used as mean±S.D., significant difference was tested by student’s *t*-test. *P<0.05, **P<0.01, ***P<0.001, *P*-value <0.05 were considered as statistically significant

We then explored the role of demethylzeylasteral in apoptosis by flow cytometry. Melanoma cells treating with 5 *μ*M demethylzeylasteral for 48 h were stained with Propidium Iodide (PI) and AnnexinV-APC. The results displayed that demethylzeylasteral induces apoptosis in melanoma cells (Figures 3a and b). To further confirm these results, we treated MV3 and A375 cells with 5 *μ*M demethylzeylasteral for 0, 12, 24, 36 and 48 h. Cleaved Caspase3 (C-Caspase3) and PARP or cleaved PARP (C-PARP) from these cells were tested and results showed that C-Caspase3 and C-PARP increased in a time-dependent manner (Figures 3c and d). Moreover, C- Caspase3 and PARP from cells treating with different concentration of demethylzeylasteral (1, 5, 10 and 20 *μ*M, DMSO was used as control) for 48 h were detected. Results showed that C-Caspase3 and C-PARP increased in a dose-dependent manner (Figures 3e and f).

### Demethylzeylasteral inhibits MCL1, whose overexpression recovers the proliferation ability inhibited by demethylzeylasteral

**Figure 4:**
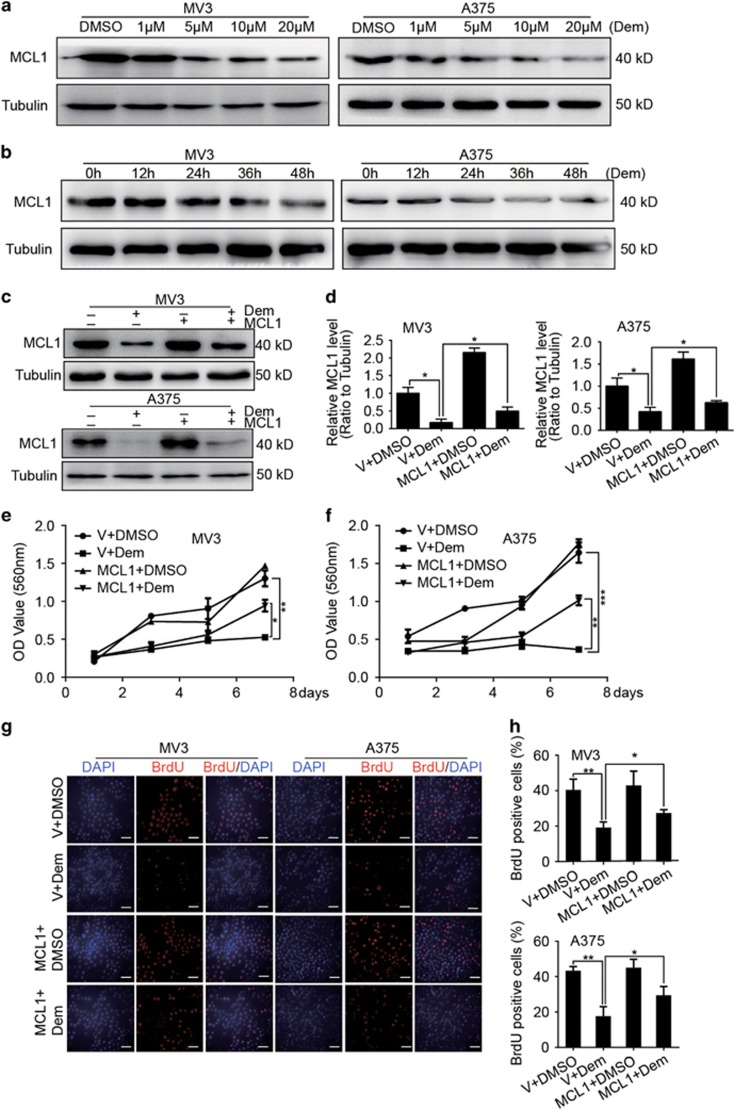
Demethylzeylasteral inhibits MCL1, whose overexpression recovers the proliferation ability inhibited by demethylzeylasteral. (**a**) The expression of MCL1 in MV3 and A375 cells treating with demethylzeylasteral in different concentration of 1, 5,10 and 20 *μ*M for 48 h. DMSO was used as control. Tubulin was used as internal reference. (**b**) The expression of MCL1 in MV3 and A375 cells treating with 5 *μ*M demethylzeylasteral for different time of 0, 12, 24 and 48 h. Tubulin was used as internal reference. (**c**) The expression of MCL1 in 5 *μ*M demethylzeylasteral-treated cells enhancing of MCL1 or empty vector. (**d**) Densitometry of western blot in panel (**c**). (**e**,**f**) Growth curve of MCL1 overexpressing MV3 and A375 cells after treating with 5 *μ*M demethylzeylasteral. DMSO and empty vector were used as control. (**g**) BrdU-positive cells in MCL1-overexpressed MV3 and A375 cells after treating with 5 *μ*M demethylzeylasteral. DMSO and empty vector were used as control. scale bar=100 *μ*m. (**h**) Quantification of BrdU-positive MV3 and A375 cells in panel (**f**). All experiments were repeated at least three times. All data were used as mean±S.D., significant difference was tested by student’s *t*-test. *P<0.05, **P<0.01, ***P<0.001, *P*-value <0.05 were considered as statistically significant

After treating with different concentration of demethylzeylasteral (1, 5, 10 and 20 *μ*M, DMSO was used as control) for 48 h, we found that an important anti-apoptotic protein MCL1, which belongs to BCL2 family, was significantly decreased in a dose-dependent manner in both MV3 and A375 cells (Figure 4a). Moreover, MV3 and A375 cells were dosed with 5 *μ*M demethylzeylasteral for 0, 12, 24, 36 and 48 h. Results indicated that MCL1 reduced in a time-dependent manner as well (Figure 4b). MV3 and A375 were then stably infected with lentivirus encoding MCL1. Western blot demonstrated that MCL1 was upregulated after infection. DMSO and empty vector were used as control (Figures 4c and d). MTT assay was employed after treating with 5 *μ*M demethylzeylasteral in MCL1- and empty vector-overexpressed melanoma cells for 48 h, DMSO and empty vector were used as control. Results indicated that overexpression of MCL1 recovered the growth curve declined by demethylzeylasteral treatment (Figures 4e and f). After treating with 5 *μ*M demethylzeylasteral for 48 h, BrdU staining was performed. Results illustrated that overexpression of MCL1 rescued the DNA synthesis reduced by demethylzeylasteral (Figures 4g and h).

### Demethylzeylasteral induces cell cycle arrest through suppressing the expression of MCL1, which interacts with CDK2 and inhibits the ubiquitin-dependent degradation of the latter

**Figure 5:**
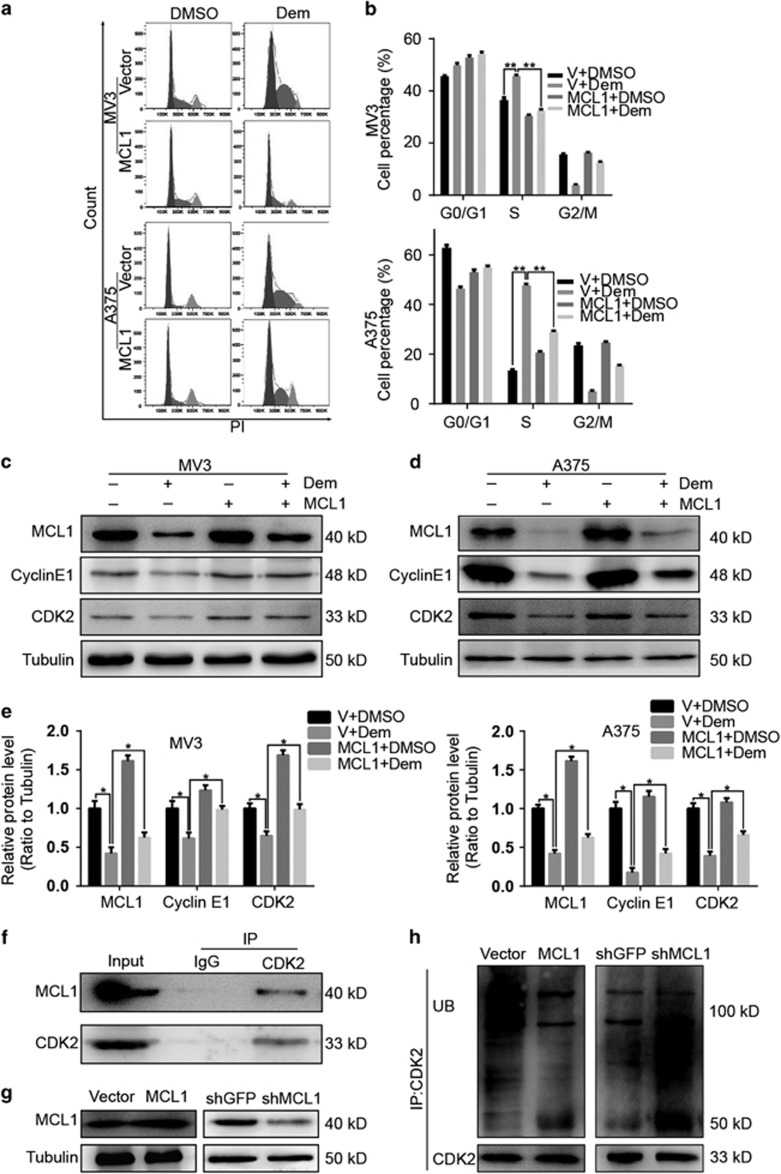
Demethylzeylasteral induces cell cycle arrest through suppressing the expression of MCL1, which interacts with CDK2 and inhibits the ubiquitin-dependent degradation of the latter. (**a**) Cell cycle in MV3 and A375 cells overexpressing with MCL1 after treating with 5 *μ*M demethylzeylasteral for 24 h. DMSO and empty vector were used as control. (**b**) Percentage of MV3 and A375 cells from panel (**a**) in different phase. (**c**,**d**) The expression of CDK2 and Cyclin E1 together with MCL1 were checked in MCL1-overexpressed MV3 and A375 cells with 5 *μ*M demethylzeylasteral treatment for 48 h. DMSO and empty vector were used as control. Tubulin was used as internal reference. (**e**) Densitometry of western blot in panel (**c**,**d**). (**f**) The interaction of MCL1 and CDK2 in A375 cells. (**g**) MCL1 was overexpressed and downregulated respectively in A375 cells. (**h**) The ubiquitination of CDK2 in A375 cells enhancing and knocking down of MCL1. All experiments were repeated at least three times. All data were used as mean±S.D., significant difference was tested by student’s *t*-test. *P<0.05, **P<0.01, ***P<0.001, *P*-value <0.05 were considered as statistically significant

In order to reveal whether downregulation of MCL1 is involved in effects induced by demethylzeylasteral, we dosed MCL1- and empty vector-overexpressed melanoma cells with 5 *μ*M demethylzeylasteral, DMSO and empty vector were used as control. After treating for 24 h, cells were harvested and cell cycle was examined using flow cytometry. Results showed that comparing with the empty vector-overexpressed group, the group overexpressing with MCL1 showed decline in percentage of S phase in both MV3 and A375cells (Figures 5a and b), which means overexpression of MCL1 rescues cell cycle arrest induced by demethylzeylasteral. Additionally, we checked the expression of CDK2 and Cyclin E1, which are essential for G1/S transition. Results are consistent with above, we observed that in comparison with the empty vector-overexpressed group, cells overexpressing with MCL1 following 5 *μ*M demethylzeylasteral treatment for 48 h showed downregulation of CDK2 and Cyclin E1 (Figures 5c–e). These results indicated the relationship between MCL1 and cell cycle. We then focused on CDK2 to explore the potential mechanism. Results showed that MCL1 interacts with CDK2 (Figure 5f). Furthermore, after enhancing and knocking down the expression of MCL1 respectively (Figure 5g), ubiquitination assay was performed. Results showed that overexpression of MCL1 inhibited the ubiquitin-dependent degradation of CDK2. In contrary, downregulation of MCL1 accelerate the ubiquitin-dependent degradation of CDK2 (Figure 5h). These results indicated that cell cycle arrest caused by demethylzeylasteral can be rescued by restoring the expression of MCL1, which worked through inhibiting ubiquitin-dependent degradation of CDK2.

### MCL1 recovery decreases apoptosis induced by demethylzeylasteral

**Figure 6:**
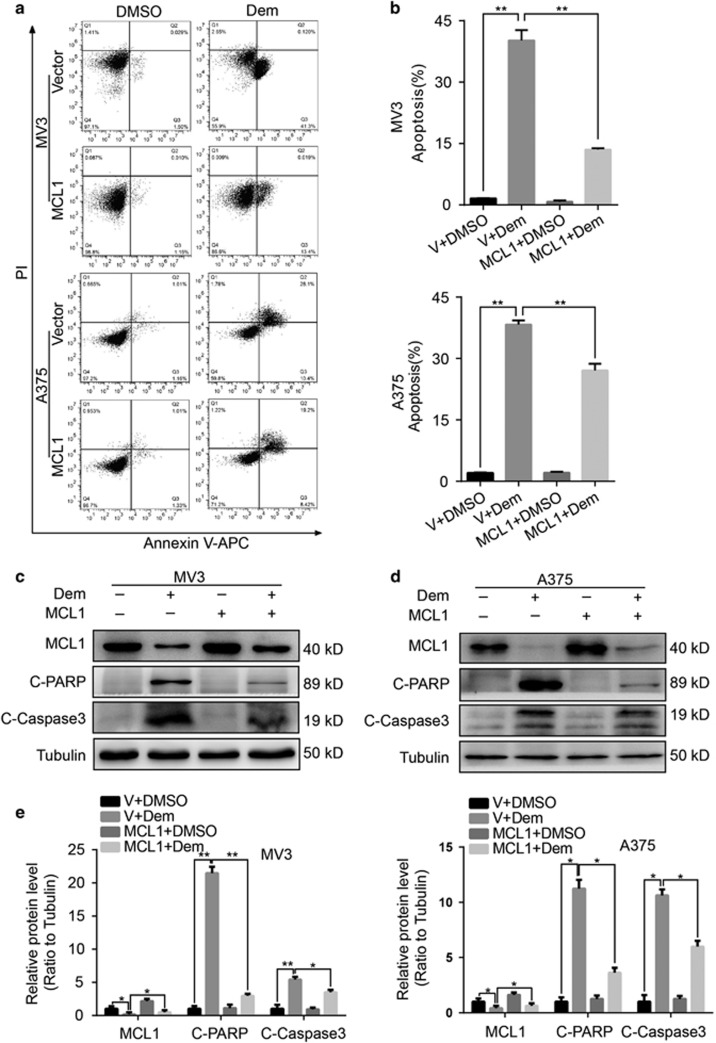
Overexpression of MCL1 turnover the apoptosis induced by demethylzeylasteral. (**a**) Apoptosis was analyzed in MV3 and A375 cells overexpressing with MCL1 after treating with 5 *μ*M demethylzeylasteral for 48 h. DMSO and empty vector were used as control. (**b**) Apoptotic rate of MV3 and A375 cells in panel (**a**) was quantified. (**c**,**d**) The expression of MCL1 and cleaved-PARP together with cleaved-Caspase3 were checked in MV3 and A375 cells overexpressing with MCL1 after treating with 5 *μ*M demethylzeylasteral for 48 h. DMSO and empty vector were used as control. Tubulin was used as internal reference. (**e**) Densitometry of western blot in panel (**c**,**d**). All experiments were repeated at least three times. All data were used as mean±S.D., *n*=3, significant difference was tested by student’s *t*-test. *P<0.05, **P<0.01, ***P<0.001, *P*-value <0.05 were considered as statistically significant

To further analyze effects of MCL1 in apoptosis caused by demethylzeylasteral, we treated melanoma cells overexpressing MCL1 and empty vector with 5 *μ*M demethylzeylasteral, DMSO and empty vector were used as control. After 48 h incubation, we harvested cells and analyzed apoptosis by flow cytometry. Results showed that apoptosis induced by demethylzeylasteral decreased in the group overexpressing with MCL1 (Figures 6a and b), which indicated that restoring the expression of MCL1 decreases apoptosis induced by demethylzeylasteral. Moreover, we dosed cells with 5 *μ*M demethylzeylasteral for 48 h and then assessed the proteins, C-caspase3 and C-PARP. Western blot revealed that comparing with empty vector-overexpressed counterpart, the expression of C-caspase3 and C-PARP were decreased in cells overexpressing with MCL1 following demethylzeylasteral treatment (Figures 6c).

### Demethylzeylasteral inhibits clonogenicity and tumorigenesis in melanoma cells through downregulating the expression of MCL1

**Figure 7:**
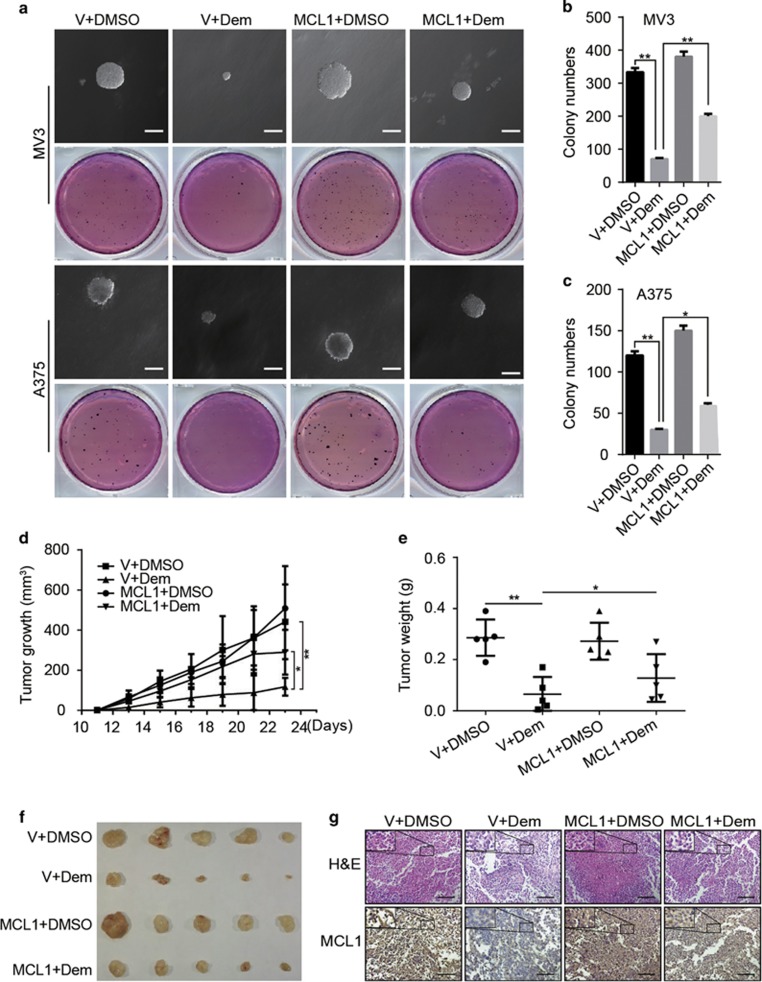
Demethylzeylasteral inhibits clonogenicity and tumorigenesis in melanoma cells through downregulating the expression of MCL1. (**a**) colony-formation ability of MV3 and A375 cells overexpressing with MCL1 after treating with 5 *μ*M demethylzeylasteral. DMSO and empty vector were used as control. scale bar=100 *μ*m. (**b**,**c**) Colony numbers in panel (**a**) were quantified. (**d**) Tumor volume of indicated mice. DMSO and empty vector were used as control. (**e**) Tumor weight of indicated mice. (**f**) Photograph of tumors from indicated mice. (**g**) H&E staining and IHC of MCL1 in indicated tumors. scale bar=100 *μ*m. All data were used as mean±S.D., *n*=3, significant difference was tested by student’s *t*-test. *P<0.05, **P<0.01, ***P<0.001, *P*-value <0.05 were considered as statistically significant

In order to know whether demethylzeylasteral affect clonogenicity in melanoma cells, we performed soft agar assay. Results displayed that in comparison with DMSO group, 5 *μ*M demethylzeylasteral group has less and smaller colonies. Additionally, overexpression of MCL1 resulted in more colonies as well as greater volume comparing with empty vector-overexpressed group (Figures 7a–c). These mean demethylzeylasteral inhibits clonogenicity of melanoma cells *in vitro* and the inhibition can be retrieved by overexpression of MCL1. Simultaneously, subcutaneously xenograft of A375 cells was performed on 4-week-old female nude mice. Results demonstrated that mice injected with demethylzeylasteral have smaller tumor volume together with smaller tumor weight comparing with mice injecting with DMSO. Furthermore, after demethylzeylasteral injection, mice transplanting with MCL1-overexpressed A375 cells showed bigger tumor volume and bigger tumor weight than mice injecting with empty vector-overexpressed A375 cells.(Figures 7d–f), which means demethylzeylasteral inhibits tumorigenesis *in vivo* and the inhibition can be retrieved by overexpression of MCL1. Hematoxylin and eosin (H&E) assay and immunohistochemical (IHC) staining with MCL1 were performed and further supported the results mentioned above (Figure 7g).

## Discussion

Demethylzeylasteral is a monomer obtained from *Tripterygium wilfordii* Hook F. Since its unveiling in the 1960 in China, accumulated investigations have demonstrated that demethylzeylasteral possesses various pharmacological activities. Over the past decades, quite a few reports mentioned the inflammation inhibition and immunosuppression properties of demethylzeylasteral.^50, 51^ There are many other inhibitory abilities of demethylzeylasteral. For instance, demethylzeylasteral was found to inhibit Ca^2+^ currents in mouse spermatogonia;^52^ Estradiol glucuronidation can be inhibited by demethylzeylasteral^53^; demethylzeylasteral exhibits inhibitory effect towards UDP-glucuronosyltransferase (UGT)^54^; demethylzeylasteral has an antifertility ability^55^. Certainly, there are also some studies indicated that demethylzeylasteral can inhibit tumor growth.^56^ Still, the specific impact together with the mechanism of the anti-tumor activity is unclear.

MCL1 is frequently amplified in numerous cancers, such as breast cancer, AML and NSCLC. As an anti-apoptotic protein belonging to BCL2 family, MCL1 has recently been regarded as an important target for cancer therapy.^36, 57^ In this study, demethylzeylasteral is proved to cause cell cycle arrest at S phase and inducing apoptosis in melanoma cells by inhibiting MCL1, which indicates that MCL1 is involved in both cell cycle and apoptosis. BCL2 family members sustain the homeostasis between cell death and survival through their anti-apoptotic and pro-apoptotic counterparts.^58^ As a pro-survival member of BCL2 family, MCL1 has an anti-apoptotic effect. Inhibiting MCL1 releases the anti-survival protein BAK and BAX from MCL1,^58^ and then accelerates apoptosis, which is consistent with our study. After demethylzeylasteral treatment, MCL1 is repressed and then induces apoptosis in melanoma cells. Nevertheless, there are few evidences between MCL1 and cell cycle. Here, we found that demethylzeylasteral did induce cell cycle arrest at S phase and by enhancing MCL1, the situation could be recovered. These results confirm the relationship between MCL1 and cell cycle. We then focused on CDK2, a key regulator in S phase.^59^ Results indicate that there is an endogenous interaction between MCL1 and CDK2. Furthermore, we found that enhancing the expression of MCL1 decreased the ubiquitinated CDK2. Conversely, knocking down of MCL1 in melanoma cells increased the ubiquitinated CDK2. These findings indicate that MCL1 inhibits the ubiquitin-dependent degradation of CDK2. Specifically, demethylzeylasteral inhibits MCL1 and then accelerates the ubiquitin-dependent degradation of CDK2, which results in cell cycle arrest at S phase. Taken together, these studies provide a new insight into the relationship of cell cycle and apoptosis. Simultaneously, this work indicates that demethylzeylasteral may act as a potential inhibitor of MCL1. Furthermore, these findings provide more information for the exploration of MCL1 inhibitors.

## Materials and methods

### Cell culture

Human melanoma cell lines, MV3 and A375 as well as human embryonic renal cell line, 293FT were obtained from American Type Culture Collection (ATCC, Manassas, VA, USA). These cells were tested mycoplasma-negative. MV3 cells were cultured in RPMI-1640 (Gibco, New York, NY, USA). Dulbecco’s modified Eagle’s medium (DMEM, Gibco) was used for maintaining A375 cells. These two medium were supplemented with 10% fetal bovine serum (FBS, Gibco) together with 1% penicillin and streptomycin (P/S, Invitrogen, Califonia, CA, USA). 293FT cells were cultured in DMEM additionally contained 1% G418 (Invitrogen), 2% glutamine (Invitrogen), 1% non-essential amino acids (Invitrogen) and 1% sodium pyruvate (Invitrogen). Cell lines mentioned above were cultured in a standard condition (5% CO_2_ at 37 °C).

### Demethylzeylasteral treatment

Demethylzeylasteral (molecular formula: C29H36O6, relative molecular mass: 480.59) was purchased from Must (Chengdu, China) and then dissolved in DMSO as 40 mM stock solution. Demethylzeylasteral was then used to treat melanoma cell lines, MV3 and A375 with different concentration (1, 5, 10 and 20 *μ*M), DMSO was used as control. Meanwhile, different time (0, 12, 24 and 48 h) exposing to 5 *μ*M demethylzeylasteral was performed as well. Morphology caused by demethylzeylasteral with different concentration (1, 5, 10 and 20 *μ*M) was detected by microscopy (Olympus, Japan); meanwhile, cell numbers were counted using hemocytometer. All experiments were performed in triplicates independently.

### Cell viability assay

MTT (Sigma Aldrich, St. Louis, MO, USA) assay was performed to investigate the cell viability.^60^ Briefly, MV3 and A375 cells in logarithmic phase were counted and seeded in 96-well plates (800 cells in 200 *μ*l medium per well) then attached overnight. RPMI-1640 and DMEM that contained demethylzeylasteral in different concentration (1, 5, 10 and 20 *μ*M) were then changed to MV3 and A375 cells respectively. DMSO was used as control. At designated time points, cells were incubated with MTT (5 mg/ml, 20 *μ*l per well) in 37 °C incubator for 2 h. DMSO (150 *μ*l) was further used to solve the formazan and then monitored the absorbance at 560 nm by microplate reader (Thermo Fisher, Waltham, MA, USA). Data were analyzed by Graphpad. All experiments were performed in triplicates independently.

### BrdU staining

Cell proliferation was monitored by BrdU staining.^61^ 2 × 10^4^ melanoma cells in logarithmic phase were seeded in 24-well plates and then attached overnight in 37 °C incubator. demethylzeylasteral (5 *μ*M) was then added to medium and changed to melanoma cells. DMSO was used as control. After 48 h, 10 *μ*g/ml BrdU (Sigma Aldrich, USA) was added into cells for 2 h and then fixed with 4% paraformaldehyde for 15 min. After treating with 2 M HCL and then treating with 0.3% TritonX-100, cells were blocking with 10% goat serum (ZSGB-Bio, Beijing, China). Cells were then successively incubated with BrdU primary antibody (Abcam, Cambridge, MA, USA) and then with secondary antibody (Life, New York, NY, USA). Before observing by microscopy, cells were stained with Hochest (Life, New York, NY, USA). BrdU-positive cells in random fields were counted.

### Flow cytometry analysis

Cells were cultured in the medium containing 5 *μ*M demethylzeylasteral and then harvested for flow cytometry analysis. DMSO was used as control. For cell cycle assay, cells treating with demethylzeylasteral were harvested at 24 h and washed with cold phosphate-buffered saline (PBS) then fixed with 75% ethanol in 4 °C for 24 h. After washing twice with PBS, cells were incubated in 200 *μ*l PBS containing 1 *μ*l PI (BD, San Jose, CA, USA) and 1 *μ*l RNaseA (Sigma Aldrich, USA) at 37 °C for 30 min. Cells were then analyzed with BD accuri C6 flow cytometry (BD, USA). For cell apoptosis, cells treating with demethylzeylasteral were collected at 48 h and washed with cold PBS twice. Cells were then incubated in 100 *μ*l binding buffer (BD, USA) containing PI (5 *μ*l) and AnnexinV-APC (BD, USA, 2.5 *μ*l) for 20 min at room temperature. Flow cytometry as well as FlowJo software were used to analyze the cell cycle and apoptosis of melanoma cells. All experiments were performed in triplicates independently.

### Western blot analysis

Cells were collected and then lysed in a RIPA lysis buffer (Beyotime, China) with Phenylmethanesulfonyl fluoride (Beyotime, Shanghai, China). Cell lysates were denatured at 100 °C for 30 min. 10% and 12% SDS-PAGE gel were used to separate proteins. Proteins were then transferred to polyvinylidene difluoride membranes. After blocking membranes in 5% bovine serum albumin at room temperature for 2 h. Primary antibodies against CDK2 (1 : 1000, Cell Signaling Technology, Beverly, MA, USA), Cyclin E1 (1 : 1000, Cell Signaling Technology, USA), PARP (1 : 1000, Cell Signaling Technology, USA; 1 : 1000, Proteintech, Chicago, IL, USA), cleaved-PARP (1 : 1000, Cell Signaling Technology, USA), cleaved-Caspase3 (1 : 1000, Cell Signaling Technology, USA), Tubulin (1 : 1000, Beyotime, China) and MCL1 (1 : 2000, Proteintech, China) were incubated with membranes at 4 °C overnight. Membranes were incubated with horseradish peroxidase-conjugated secondary antibody (HRP-conjugated secondary antibodies (goat anti-mouse IgG and goat anti-rabbit IgG, 1 : 10 000, Beyotime, China) at room temperature for 2 h. Proteins were finally visualized by ECL system (Beyotime, China) and then captured by ProXima chemiluminescence gel imaging system (Isogen, De Meern, Utrecht, Northerlands).

### Transfection and infection

Vector encoding human MCL1 was purchased from YouBio (Changsha, China). The pCDH-CMV-MCS-EF1-copGFP vector was obtained from Addgene (Beijing, China) and kept in our laboratory. MCL1 short hairpin RNA (shRNA) and GFP shRNA were purchased from Sigma-Aldrich and then cloned into the pLKO.1 vector. For stable transfection, 293FT cells were prepared to 90% density and then plasmids including pCDH-CMV-MCS-EF1-copGFP-MCL1, empty vector, GFP shRNA or MCL1 shRNA and packaging plasmids (pLP1, pLP2, pLP/VSVG) were collectively transfected into 293FT cells using ViaFect transfection reagent (Promega, Madison, WI, USA) according to the manufacturer’s instruction. Viral supernatants were collected after 48 h and then infected into the melanoma cells helping with Polybrene. Cells were then selected with puromycin and passaged.^62^ GFP shRNA and empty vector were used as control.

### Soft agar assay

Soft agar assay was used to detect colony formation ability of melanoma cells. 1.5 ml 2 × RPMI-1640 (Gibco) and 2 × DMEM (Gibco) containing 0.6% agarose (Sigma-Aldrich, USA) were added to six-well plates as base agar. Eight hundred cells in logarithmic phase mixing with medium containing 0.3% agar as well as 5 *μ*M demethylzeylasteral were added onto base agar. After culturing at 37 °C incubator for 21 days, colonies were captured by microscopy and counted after staining with MTT.

### Tumor xenografts

All animal experiments were approved by the animal ethics committee of Southwest University and performed humanly in accordance with guidelines of the Care and Use of Laboratory Animals (Ministry of Science and Technology of China, 2006). Four-week-old female nude mice were purchased and housed in SPF room. 1 × 10^6^ cells infected with MCL1 and empty vector in 100 *μ*l basic medium were subcutaneously injected to flanks of mice, respectively (cells overexpressed with empty vector were on the left side, cells infected with MCL1 on the right). After administering the injection for 1 week, the mice were randomly divided into two groups. One was then injected with demethylzeylasteral (5 mg/kg) and the other was injected with DMSO as control, each group was injected with demethylzeylasteral or DMSO every 2 days for 14 days. During this period, the body weight as well as tumor volume (tumor volume= (*π*/6) × length × width^2^) were measured every 2 days.^62^

### Immunoprecipitation (IP) and ubiquitination assay

Cells were incubated with proteasome inhibitor MG132 (50 *μ*g/ml, Selleck, Houston, TX, USA) for 6 h in 37 °C incubator and then lysed with IP lysis buffer.^63^ CDK2 antibody (1 *μ*g, Cell Signaling Technology) as well as IgG (rabbit) was then incubated with cell lysate to pull down the CDK2 proteins. After washing with PBS for five times, cells were denatured at 100 °C for 30 min and then separated by 10% SDS-PAGE gel. MCL1 antibody was used to check the interaction between MCL1 and CDK2. Ubiquitin antibody was used to detect the ubiquitination of CDK2.

### Recover assay

Cells were transfected with the vector overexpressing with MCL1 or GFP. Cells stably expression of MCL1 were exposed to 5 *μ*M demethylzeylasteral and then harvested to investigate the proliferation together with apoptosis by MTT, BrdU, flow cytometry, western blot, tumor xenografts and soft agar. Cells stably expressing empty vector were used as control.

### Statistics analysis

Graphpad were used for statistics analysis. Cell cycle together with apoptosis was analyzed by FlowJo. Quantitative data were presented as mean±S.D. (standard deviation). Significant difference was computed by student’s *t*-test. *P<0.05, **P<0.01, ***P<0.001, *P*-value<0.05 were considered as statistically significant.

## Publisher’s Note

Springer Nature remains neutral with regard to jurisdictional claims in published maps and institutional affiliations.
